# Analysing the surface morphology of annealed FTO/ZnS bilayer films: stereometric, fractal, and wettability approaches

**DOI:** 10.1038/s41598-024-65118-w

**Published:** 2024-06-20

**Authors:** Leila Eftekhari, Mohsen Ghasemi

**Affiliations:** 1 Department of Physics, Faculty of Sciences, Bu Ali Sina University, P.O. Box 65174, Hamedan, Iran; 2 Department of Physics, Faculty of Sciences, Shahrekord University, P.O. Box 115, Shahrekord, Iran; 3 Nanotechnology Research Institute, Shahrekord University, Shahrekord, 8818634141 Iran

**Keywords:** Advanced fractal parameters, Atomic force microscope, FTO/ZnS bilayers, Microtexture, Stereometric analysis, Wettability, Statistical physics, Surfaces, interfaces and thin films

## Abstract

The surface micromorphology and roughening of the thermal evaporation-coated FTO/ZnS bilayer thin films annealed at 300, 400, 500, and $550\,^\circ{\text{C}}$ for 1 h have been studied. AFM images of the prepared samples were analysed by the MountainsMap software, and the effects of the annealing temperature on the surface texture of the FTO/ZnS thin film’s surface were investigated. Stereometric and advanced fractal analyses showed that the sample annealed at $500\,^\circ{\text{C}}$ exhibited greater surface roughness and greater skewness and kurtosis. This film also has the most isotropic surface and exhibits the highest degree of heterogeneity. Also, despite the decrease in surface roughness with increasing temperature from 500 to $550 \,^\circ{\text{C}}$, the fractal dimension tends to increase. The static water contact angle measurements indicate that the film annealed at $500 \,^\circ{\text{C}}$ exhibits higher hydrophobicity, which can be attributed to its greater topographic roughness. Our research indicates that the surface morphology of FTO/ZnS bilayer thin films is influenced by the annealing temperature. Changing factors such as roughness, fractality, and wettability parameters to help improve surface performance make the FTO/ZnS bilayer suitable for application in electronic and solar systems.

## Introduction

Compared to bulk materials, nanoparticles, and thin films exhibit fantastic properties^1^. Currently, owing to their distinct physical attributes, which contribute to practical applications in domains such as flat panel displays^2^, solar systems^3^, sensors^4^, alpha-particle detectors^5^, and light-emitting diodes (LEDs)^6^, zinc sulfide (ZnS) thin films have attracted significant scientific interest. Consequently, various methodologies have been employed to achieve the nanomaterials and thin films’ desired composition and structures. These approaches include spray pyrolysis^7^, sputtering^8^, thermal evaporation^9^, sol-gel, and electrodeposition^10^ techniques. Due to its high refractive index (2.35), ZnS has excellent coating and antireflective properties^11^. Additionally, ZnS has a wide bandgap energy of 3.7 eV at room temperature^12^ and low optical absorption in the visible and infrared regions^13^. Some work on ZnS thin films has been reported in the literature in the field of solar cells^14^ or considering only the wettability^15^ or fractal properties^16^. Farazin et al. documented some optical morphological and structural characteristics and contact angle measurements of ZnS-scotch tape nanocomposite materials^17^. On the other hand, fluorine-doped SnO$_2$ (FTO), as transparent conductive oxides (TCOs), is one of the materials that attracted attention due to its excellent chemical inertness, thermal stability, mechanical hardness, and its use in various fields. FTO thin films are suitable for having controlled morphology, including roughness and surface structure^18,19^. Adding certain amounts of fluorine to SnO$_2$ as a dopant will enhance its structural, optical, and electrical properties by reducing resistance and improving crystallinity. These improvements make F-doped SnO$_2$ suitable as a window layer in solar cells and various optoelectronic devices, such as flat plate collectors and photo-thermal conversion systems^20,21^. Several previous studies have focused on surface dynamics analysis of thin films. In recent years, there has been growing interest in researching nanoscale stereometrics and conducting statistical analysis to investigate the impact of the manufacturing process on surface morphology, as demonstrated in a study by Cybo et al.^22^ on acetabular cups for endoprotheses and S. Stach et al.^23^ on Cu/Fe NP thin films. Currently, researchers are highly interested in fractal geometry because it is useful in many areas, such as medicine^24^, the food industry (quality control)^25^, face image fusion (human authentication)^26^, and concrete mesostructure^27^. Additionally, fractal-based methods have been used for the accurate characterization of surface topography^28^. Examining contact angles (CAs) and utilizing techniques such as fractal analysis and statistical analysis can offer valuable insights into the texture and wettability of surfaces^29,30^. The wetting of solid surfaces by liquids, especially water, is a fundamental and ubiquitous phenomenon with significant implications for daily life and industrial applications^31^. Since the bilayer thin film system offers advantages such as controlled interfaces, tunable properties (like conductivity, permeability, or surface energy), and enhanced performance compared to single-layer films, are utilized in structural studies to investigate material interface interactions for optimizing properties and designing new materials^32,33^. For example, the incorporation of a bilayer composed of both n- and p-type semiconductors featuring a p–n junction at the interface is a promising approach for enhancing the sensitivity and response time of gas sensor devices or magnetic recording memory devices, and the coupling effects leading to the giant magnetoresistance (GMR) phenomenon can be effectively induced by introducing a non-magnetic interface between two identical magnetic layers^34^. FTO/ZnS bilayer systems exhibit transparency and chemical stability, making them suitable for applications in solar cells, touchscreens, and electrochromic devices. In addition, their compatibility with large-scale fabrication processes makes them cost-effective for commercial production^35^. The correlation between the fractal, morphological, and wettability characteristics of the FTO/ZnS bilayer system has not yet been thoroughly investigated. Since the distinct studies have reported different values for the amount of fluorine doping, the same optimum value has not been achieved. Therefore, we typically considered about 10 wt% F-doped SnO$_2$ to form a bilayer with the ZnS film. The main goal of our work is to analyse in detail the advanced stereometric and microtexture of the FTO/ZnS bilayer thin films. We employed an atomic force microscope (AFM) to conduct surface statistical analysis, allowing us to study the impact of texture complexity on fractal and wettability properties. Also, the dependence of the water contact angle of FTO/ZnS films on roughness and annealing temperature was investigated.

## Experimental details

### Sample preparation

The FTO/ZnS bilayer system was fabricated on a glass substrate. For this purpose, the glass substrate was washed with soap and water. Then, using an ultrasonic apparatus, each sample was washed in ethanol, methanol, dichloromethane, isopropanol, acetone, and deionized water for 5 min and dried under a nitrogen flow. Preparation of the F-doped SnO$_2$ (FTO) solution was conducted as follows: First, 5.93 mmol of SnCl$_2$.2H$_2$O was dissolved in 20 ml of ethanol in a closed container. The solution was continuously stirred until all the SnCl$_2$.2H$_2$O was completely dissolved in ethanol. Then, 5.26 mmol of NH4F was dissolved in 20 ml ethanol, in another container. After stirring thoroughly, both solutions were mixed at room temperature. The combined solution was allowed to stir for 3 h to ensure complete mixing, and the resulting solution became homogeneous and stable. Next, the final solution was sprayed onto glass substrates when set on a rotating hot plate. The substrate temperature and solution flow rate were maintained at $300 \,^\circ{\text{C}}$ and 1 ml/min, respectively. The distance between the nozzle and the substrate was approximately 25 cm. The thickness of the fluorine-doped SnO$_2$ film estimated by a Dektak profilometer was about 192 nm. For the deposition of the second layer, ZnS material was purchased from Sigma Aldrich as a source material with a purity of $99.99\%$. A tantalum crucible was applied to evaporate the target material in the thermal evaporation device. The base pressure of the vacuum chamber was $9 \times 10^{-6}$ mbar, and the pressure during the deposition process was $4.1 \times 10^{-5}$ mbar. We controlled the thickness and deposition rate and measured values of 200 nm and 0.1 nm/s, respectively, with a quartz crystal system. Also, the substrate temperature was kept at room temperature during the growth process. Annealing was performed to stabilize the layers after deposition. The bilayer system was annealed in a thermal annealing furnace at 300, 400, 500, and $550 \,^\circ{\text{C}}$ for 90 min and labeled S300, S400, S500, and S550, respectively.

### Characterization

To determine the exact doping level, Micro X-ray fluorescence (XRF) analysis was performed to study the elemental composition of the F-doped SnO$_2$ powder, using the M4 Tornado model (Bruker, Germany). The XRF results showed that various elements including Sn (70.86 wt%), O (18.97 wt%), F (9.86 wt%), and traces of other impurities such as Ca (0.083 wt%), Zn (0.061 wt%), k (0.079 wt%), Fe (0.039 wt%) and Al (0.048 wt%) were identified in the sample. Topographic images were obtained using atomic force microscopy (AFM) in tapping mode/non-contact mode with the Park NX-10 model (Park Systems, South Korea). The AFM measurements were carried out at room temperature using cantilevers with a 10 nm silicon tip radius. The $3\,\upmu{\text{m}} \times 3 \,\upmu{\text{m}}$ and $256 \times 256 pixel$ AFM images were obtained with scan rates of $1.0 \; Hz$. The relative humidity (RH) and temperature were monitored using a digital hygrometer/thermometer inside the AFM chamber. The amount of relative humidity was constant during the characterization process. Measurements were performed at room temperature ($22\pm 1 \,^\circ{\text{C}}$) and relative humidity ($45\pm 1\%$). The surface wettability of the films was measured with a contact angle (CA) apparatus using a water droplet volume of about $4 \mu L$ with the SEO model (South Korea).

### Data processing and surface analysis

The commercial software MountainsMap ®premium ver.10.1.10571 (Digital Surf, Besançon, France)^36^ according to the standard ISO 25178-2:2012 was used for data processing and presentation of much of the results in this study. MountainsMap is a powerful metrological tool for surface texture analysis. Additionally, algorithms for computing advanced fractal parameters were developed in MATLAB via the box-counting method.

**Figure 1:**
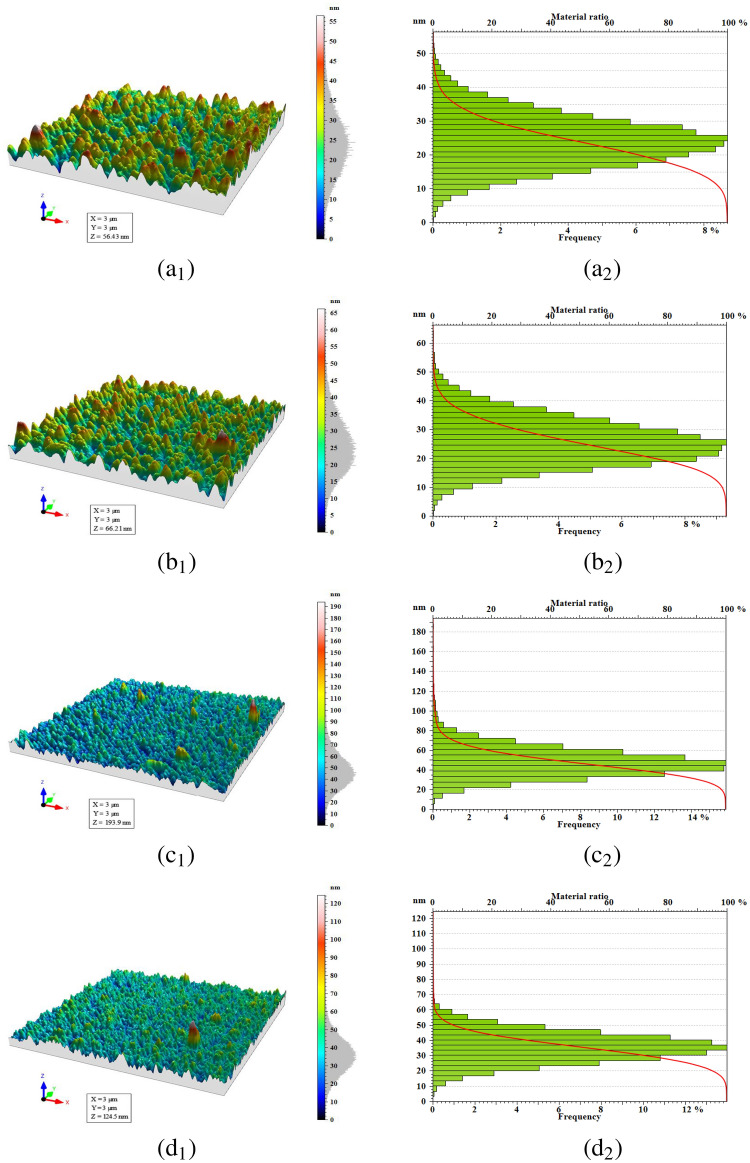
3D surface topography (index 1) and corresponding height histogram (index 2) of FTO/ZnS thin films annealed at (**a**) 300, (**b**) 400, (**c**) 500, and (**d**) $550\,^\circ{\text{C}}$.

## Image analysis and results

### Advanced stereometric analysis

Stereometric analysis has been extensively applied across distinct fields, such as biology^37^, medicine^38^, materials science, and geology. It quantitatively measures the volume, surface area, length, and other structural parameters of intricate three-dimensional objects. Stereometric parameters were employed to assess the correlation between the topographic patterns and microtextures of the film surface^39^. The 3D-AFM images in Fig. 1(a$_1$–d$_1$) show that different rough surfaces are formed due to different annealing temperatures. Moreover, Table 1 summarizes the statistical parameters deduced from $3 \,\upmu{\text{m}} \times 3 \,\upmu{\text{m}}$ scanning square areas of bilayer FTO/ZnS AFM images. The maximum values of the root mean square of height (S$_q$) and mean roughness (S$_a$) were observed in sample S500, with values of $15.21 \pm 0.78$ nm and $11.51 \pm 0.40$ nm, respectively. Furthermore, by increasing the annealing temperature from 300 to $500\,^\circ{\text{C}}$, S$_q$ increases, and then S$_q$ decreases at an annealing temperature of $550\,^\circ{\text{C}}$. This happens because the annealing process involves the interaction of various factors, such as grain growth, island coalescence, and crystallization^40^. A similar roughness trend, maximum peak height and valley depth, S$_p$, and S$_v$, and sum of the maximum height, S$_z,$ were observed for sample S500. This result suggests an increase in the spatial complexity of sample S500, specifically emphasizing higher topographic irregularity. The positive value of surface skewness (S$_{sk}$) affirms the dominance of the peak. The surface kurtosis (S$_{ku}$) displays different shapes of height distributions for samples S300 and S400 compared with samples S500 and S550. A kurtosis above three (S$_{ku}>3$) indicates a spiky or sharply peaked surface which is visible for all samples^41^. The maximum and minimum values of S$_{ku}$ in samples S500 ($8.259 \pm 0.405$) and S300 ($3.119 \pm 0.129$), respectively. Figure 1(a$_2$–d$_2$) presents the height distribution and Abbott–Firestone curve associated with relevant analysed samples. Functional parameters pertain to the distribution of heights and the cumulative curve, commonly recognized as the Abbott-Firestone curve. These parameters serve the purpose of characterizing the functional behavior of a surface, particularly in contexts such as wear, lubrication, and contact. They operate globally, encompassing field parameters, and can be viewed as statistical metrics. Owing to their association with height distribution, bearing ratio parameters can be readily extended to surfaces.

The depth histogram illustrates the density distribution of surface data points. The horizontal axis represents the bearing ratio (in %), while the vertical axis indicates the depth, accompanied by the Abbott-Firestone curve. The histogram associated with a sample annealed at $500\,^\circ{\text{C}}$ has a normal distribution, whereas samples annealed at 300 and $400\,^\circ{\text{C}}$ have a left-skewed distribution.

The Abbott-Firestone curve represents the bearing ratio curve, signifying, for a specific depth, the percentage of material traversed relative to the covered area. This function serves as the cumulative function of the amplitude distribution function. The Abbott-Firestone curves of samples S300 and S400 exhibit an approximately S-like shape, implying that the surfaces are influenced by a height distribution that approximates Gaussian characteristics, making the surfaces more uniform^42^. The arithmetic mean peak curvature (S$_{pc}$) is greater for S500, revealing that this surface has rough peaks with more rounded shapes.

**Table 1:** The height and statistical parameters of the FTO/ZnS thin films, according to ISO 25178.

	S300	S400	S500	S550
*Height Parameters*				
$S_q$-Root mean square height (nm)	$7.53\pm 0.30$	$8.05 \pm 0.39$	$15.21 \pm 0.78$	$9.78 \pm 0.45$
$S_a$-Arithmetic mean height (nm)	$5.98 \pm 0.22$	$6.44 \pm 0.20$	$11.51 \pm 0.40$	$7.67 \pm 0.32$
$^*S_{sk}$-Skewness (–)	$0.248 \pm 0.012$	$0.353 \pm 0.014$	$1.133 \pm 0.058$	$0.411 \pm 0.023$
$S_{ku}$-Kurtosis (–)	$3.119 \pm 0.129$	$3.151 \pm 0.135$	$8.259 \pm 0.405$	$5.252 \pm 0.187$
$^*S_p$-Maximum peak height (nm)	$32.36 \pm 2.10$	$40.38 \pm 2.16$	$146.02 \pm 8.57$	$88.80 \pm 5.35$
$S_v$-Maximum pit height (nm)	$24.07 \pm 0.85$	$25.83 \pm 0.84$	$47.93 \pm 1.58$	$35.75 \pm 1.45$
$S_z$-Maximum height (nm)	$56.43 \pm 2.86$	$66.21 \pm 3.20$	$193.95 \pm 6.05$	$124.5 \pm 4.41$
*Functional Parameters (Bearing ratio)*				
$S_{mr}$-Areal Material Ratio (%)	$100 \pm 0$	$100 \pm 0$	$100 \pm 0$	$100 \pm 0$
$S_{mc}$ -Inverse Areal Material Ratio (nm)	$9.80 \pm 0.31$	$10.71 \pm 0.45$	$18.51 \pm 0.72$	$12.28 \pm 0.38$
$S_{dc}$ -Inverse Areal Material Ratio (nm)	$19.26 \pm 0.86$	$20.53 \pm 0.84$	$36.07 \pm 1.30$	$24.56 \pm 1.08$
*Functional parameters (Stratified surfaces)*				
$S_{k}$-Thickness of the core (nm)	$19.16 \pm 0.85$	$20.70 \pm 1.71$	$35.18 \pm 1.86$	$24.53 \pm 1.13$
$S_{pk}$-Peak height above the core (nm)	$8.33 \pm 0.42$	$9.71 \pm 0.46$	$21.41 \pm 1.21$	$10.62 \pm 0.62$
$S_{vk}$-Valley depth below the core (nm)	$6.04 \pm 0.19$	$5.83 \pm 0.24$	$10.49 \pm 0.38$	$8.50 \pm 0.32$
$S_{mrk1}$-Peak material portion (%)	$10.98 \pm 0.33$	$11.78 \pm 0.48$	$12.39 \pm 0.48$	$10.21 \pm 0.45$
$S_{mrk2}$-Peak valley portion (%)	$90.91 \pm 4.20$	$92.44 \pm 4.54$	$91.71 \pm 4.83$	$90.19 \pm 5.14$
*Functional parameters (Volume)*				
$V_{m}$-Material Volume $(\mu m^3/\mu m^2)$	$4.11E-4 \pm 0.16E-4$	$4.39E-4 \pm 0.14E-4$	$10.88E-4 \pm 0.50E-4$	$5.27E-4 \pm 0.19E-4$
$V_{v}$-Void Volume $(\mu m^3/\mu m^2)$	$102.1E-4 \pm 4.5E-4$	$111.4 \pm 4.3E-4$	$196.0E-4 \pm 6.3E-4$	$128.0E-4 \pm 5.3E-4$
$V_{mp}$-Material volume in the hill region $(\mu m^3/\mu m^2)$	$4.11E-4 \pm 0.19E-4$	$4.39E-4 \pm 0.18E-4$	$10.88E-4 \pm 0.36E-4$	$5.27E-4 \pm 0.21E-4$
$V_{mc}$-Material volume within the core $(\mu m^3/\mu m^2)$	$67.88E-4 \pm 3.17E-4$	$71.82E-4 \pm 3.01E-4$	$124.70E-4 \pm 4.90E-4$	$86.56E-4 \pm 3.57E-4$
$V_{vc}$-Void volume within the core $(\mu m^3/\mu m^2)$	$94.7 \pm 5.0E-4$	$104.0E-4 \pm 6.0E-4$	$182.7 \pm 8.5E-4$	$117.7E-4 \pm 6.0E-4$
$V_{vv}$-Void volume below the core $(\mu m^3/\mu m^2)$	$7.47E-4 \pm 0.46E-4$	$7.44E-4 \pm 0.43E-4$	$13.28E-4 \pm 0.68E-4$	$10.30E-4 \pm 0.61E-4$
*Feature parameters*				
$S_{pd}$-Peak Density $(1/\mu m^2)$	$31.33 \pm 1.18$	$12.00 \pm 0.43$	$15.44 \pm 0.64$	$17.78 \pm 0.58$
$S_{pc}$-Arithmetic Mean Peak Curvature $(1/\mu m)$	$110.3 \pm 4.6$	$130.0 \pm 5.4$	$147.0 \pm 7.8$	$131.5 \pm 5.6$
*Hybrid parameters*				
$S_{dq}$-Void volume within the core (–)	$0.228 \pm 0.007$	$0.300 \pm 0.012$	$0.867 \pm 0.028$	$0.612 \pm 0.025$
$^*S_{dr}$-Void volume below the core (%)	$2.54 \pm 0.11$	$4.32 \pm 0.18$	$28.20 \pm 0.91$	$16.31 \pm 0.64$
*Furrows*				
*MFD*-Maximum furrow depth (*nm*)	$31.74 \pm 1.65$	$29.99 \pm 1.61$	$64.19 \pm 2.95$	$37.15 \pm 1.94$
*AFD*-Average furrow depth (*nm*)	$14.13 \pm 0.63$	$15.11 \pm 0.61$	$26.97 \pm 1.30$	$16.59 \pm 0.58$
$AFD_{sty}$-Average furrow density (cm/cm$^2$)	$75349 \pm 2291$	$80264 \pm 2849$	$86163 \pm 2783$	$85641 \pm 3220$
*Texture*				
$S_{al}$-Autocorrelation length $(\mu m)$	$0.1109 \pm 0.0051$	$0.0817 \pm 0.0035$	$0.0648 \pm 0.0020$	$0.0672 \pm 0.0025$
$^*S_{td}$-Texture direction (deg)	$132.50 \pm 8.10$	$172.20 \pm 10.44$	$19.50 \pm 1.07$	$50.74 \pm 2.85$
$S_{tr}$-Texture aspect ratio (–)	$0.8955 \pm 0.0482$	$0.8120 \pm 0.0420$	$0.9406 \pm 0.483$	$0.8454 \pm 0.0457$
$S_{sw}$-Texture aspect ratio $(\mu m)$	$0.0127 \pm 0.001$	$0.0212 \pm 0.001$	$0.0170 \pm 0.001$	$0.0170 \pm 0.001$

*Samples without significant difference according to one-sample t-test: *p*-value $$ > 0.05$$

**Figure 2:**
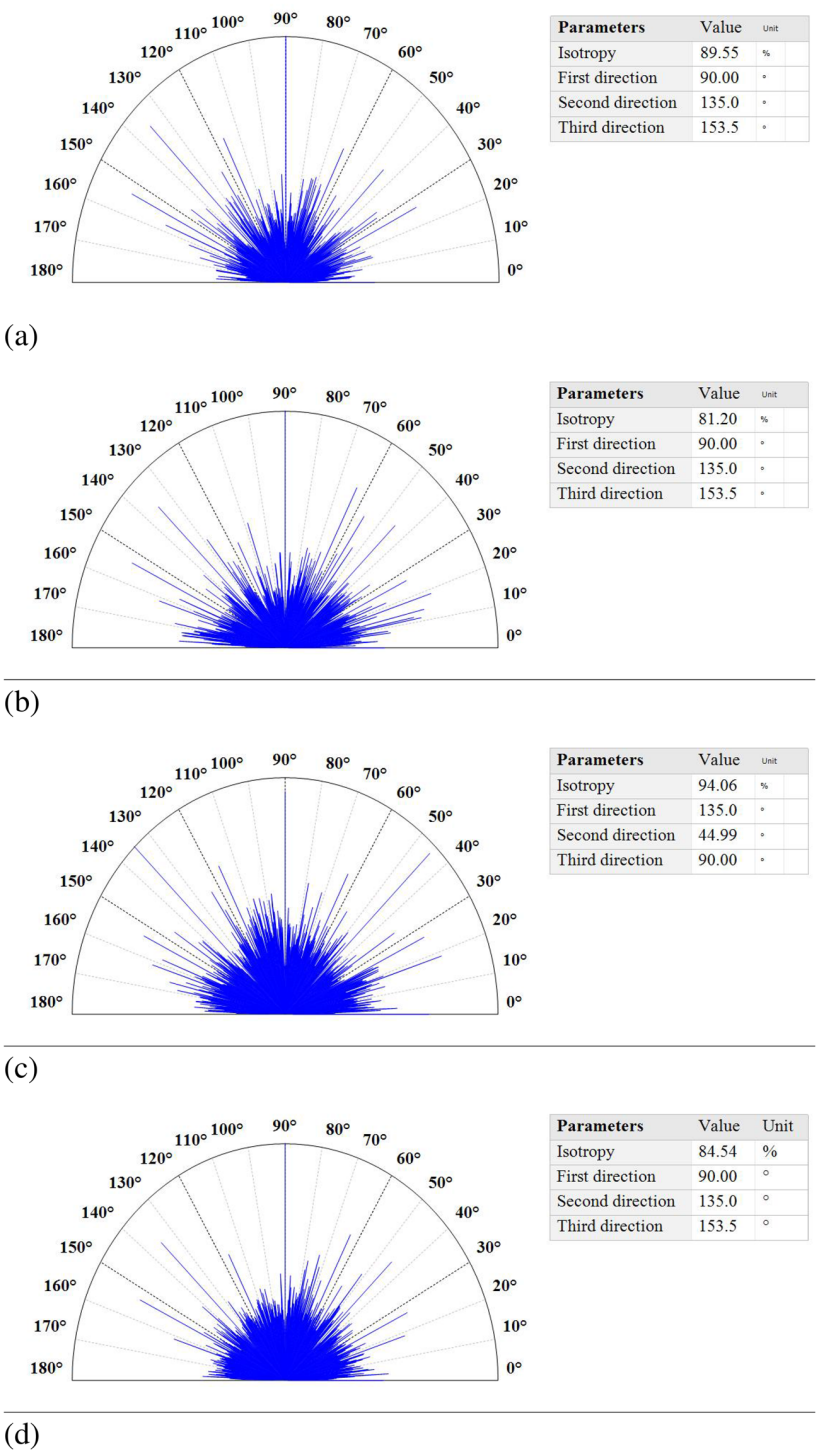
Texture direction of FTO/ZnS thin films annealed at (**a**) 300, (**b**) 400, (**c**) 500, and (**d**) $550\,^\circ{\text{C}}$.

The polar spectrum graphs in Fig. 2 represent the texture directions, whose corresponding results for each sample are summarized in the table on the right-hand side of the figure. The highest and lowest values of the isotropy parameter are related to sample S500 ($94.06\%$) and sample S400 ($81.20\%$), respectively. The first, second, and third directions are similar for samples S300, S400, and S550 ($90.00^\circ , 135.00^\circ ,$ and $153.50^\circ ,$ respectively). Sample S500 exhibited the highest value in the first direction ($135.00^\circ$) and the lowest values in the second and third directions ($44.99^\circ$, $90.00^\circ$).

**Figure 3:**
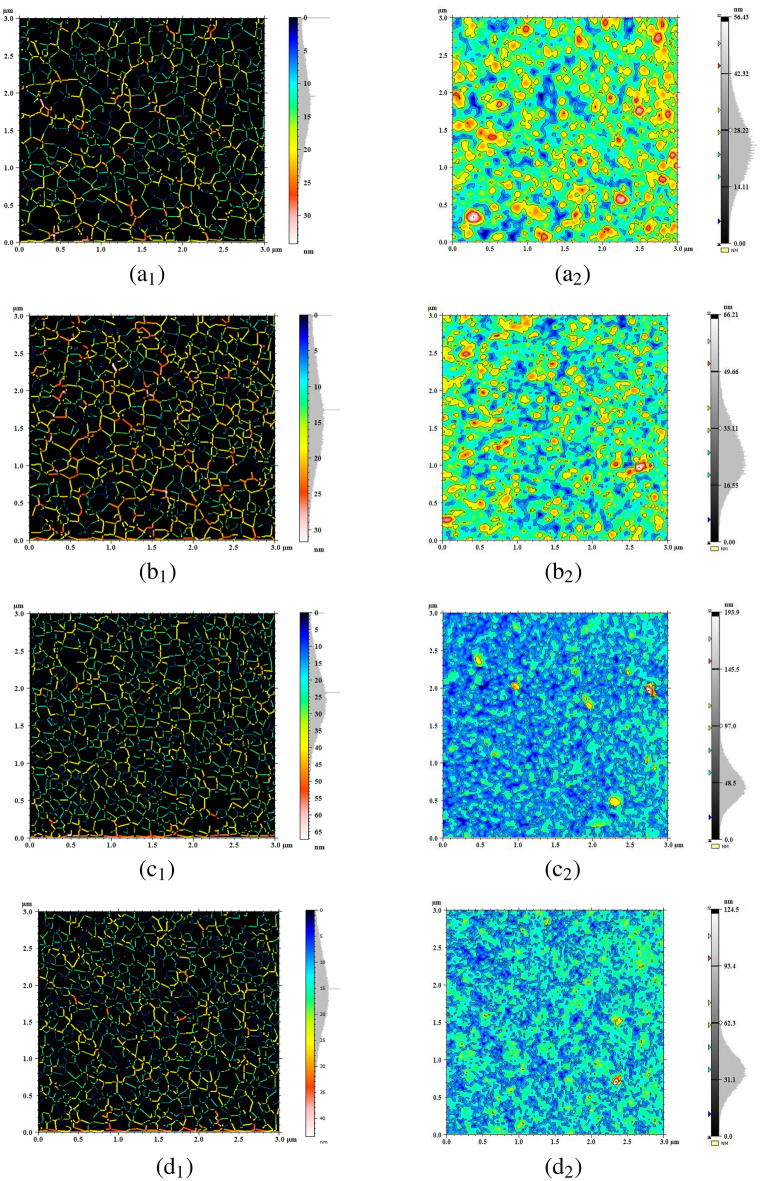
Qualitative rendering of furrows (index 1) and contour lines (index 2) of FTO/ZnS thin films annealed at (**a**) 300, (**b**) 400, (**c**) 500, and (**d**) $550\,^\circ{\text{C}}$.

**Figure 4:**
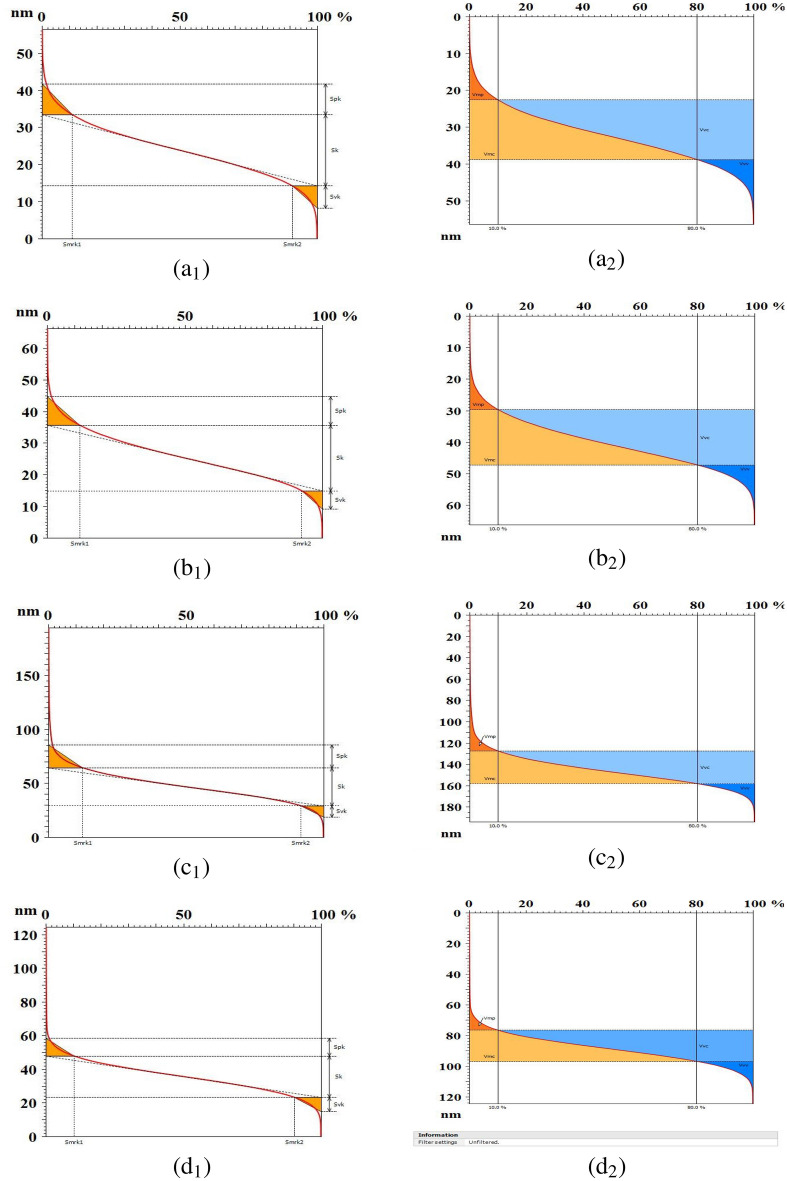
Functional (stratified) parameters (index 1) and active (volume) parameters (index 2) based on the Abbott-Firestone curve of FTO/ZnS thin films annealed at (**a**) 300, (**b**) 400, (**c**) 500, and (**d**) $550\,^\circ{\text{C}}$.

This study was performed to analyse the surface complexity of the samples. One approach to emphasize the image texture involves representing the 3D surface of thin films. More sophisticated qualitative rendering methods are available to highlight surface texture and simulate flooded images using contour lines and microfurrows. The flow area and fluid entrance are depicted by the microfurrows generated on the bilayer FTO/ZnS surfaces (see Fig. 3(a$_1$–d$_1$)). The furrow images of all the samples are uniform. Lighter areas display rougher furrows, while darker ones display valleys. The quantitative features of the furrow map, including the maximum furrow depth, average furrow depth, and average furrow density, were extracted and are presented in Table 1. The higher maximum furrow depth was observed in sample S500 ($64.19 \pm 2.95$ nm), suggesting the greatest roughness of the S500 surface. Sample S500 also shows the same behavior for the average furrow depth (AFD) and average furrow density (AFD$_{sty}$) with values of $26.97 \pm 1.30$ nm and $86163 \pm 2783$ cm/cm$^2$, respectively. The approximate similarity of the depth of the channels for samples S500 and S550 shows that the samples have almost the same wettability. This also applies to samples S300 and S400. The high-quality photo simulation of the flooded image through the contour lines is presented in Fig. 3(a$_2$–d$_2$). This behavior illustrates the association between the microtexture and annealing temperature. Lighter regions correspond to rougher furrows, whereas darker regions are associated with valleys^43^. Figure 4(a$_1$–d$_1$) shows Abbott-Firestone curves of thickness parameters that are connected to the core of the surface’s microtexture. The other quantitative values of the S$_k$ parameters are listed in Table 1. The highest thickness of the surface central part in the z-direction, core height (S$_k$), belonged to sample S500 ($35.18 \pm 1.86$ nm), while sample S300 presented the lowest value of $19.16 \pm 0.85$ nm. The S500 and S400 samples exhibit the highest ($10.49 \pm 0.38$ nm) and lowest ($5.83 \pm 0.24$ nm) values for reduced peak depth (S$_{vk}$). In addition, the average value of the peak/valley material portion (*Smr*1/ *Smr*2) is the percentage of the material ratio curve that corresponds to the upper/lower limit of the roughness core profile S$_k$, which does not fluctuate. The height parameters have broad applicability and are frequently employed in practical applications, specified in units of *nm* or $\mu m$. Conversely, volume parameters are infrequently utilized in the context of surface wettability studies. It seems that the void volume can be a suitable parameter for characterizing the extent of bubbles on surfaces. This choice is grounded in the understanding that the void volume parameter indicates the amount of air that would occupy the surface (normalized to the measurement area) within the specified material ratio values. These void volume measurements are derived from the surface roughness parameter, S$_a$, described in detail as a function-related parameter in^28^. Figure 4(a$_2$–d$_2$) shows the Abbott–Firestone curve, which encompasses all volume-related parameters obtained through ISO 25178:2012 (Table 1). All functional parameters (volume) had the highest values for sample S500.

**Figure 5:**
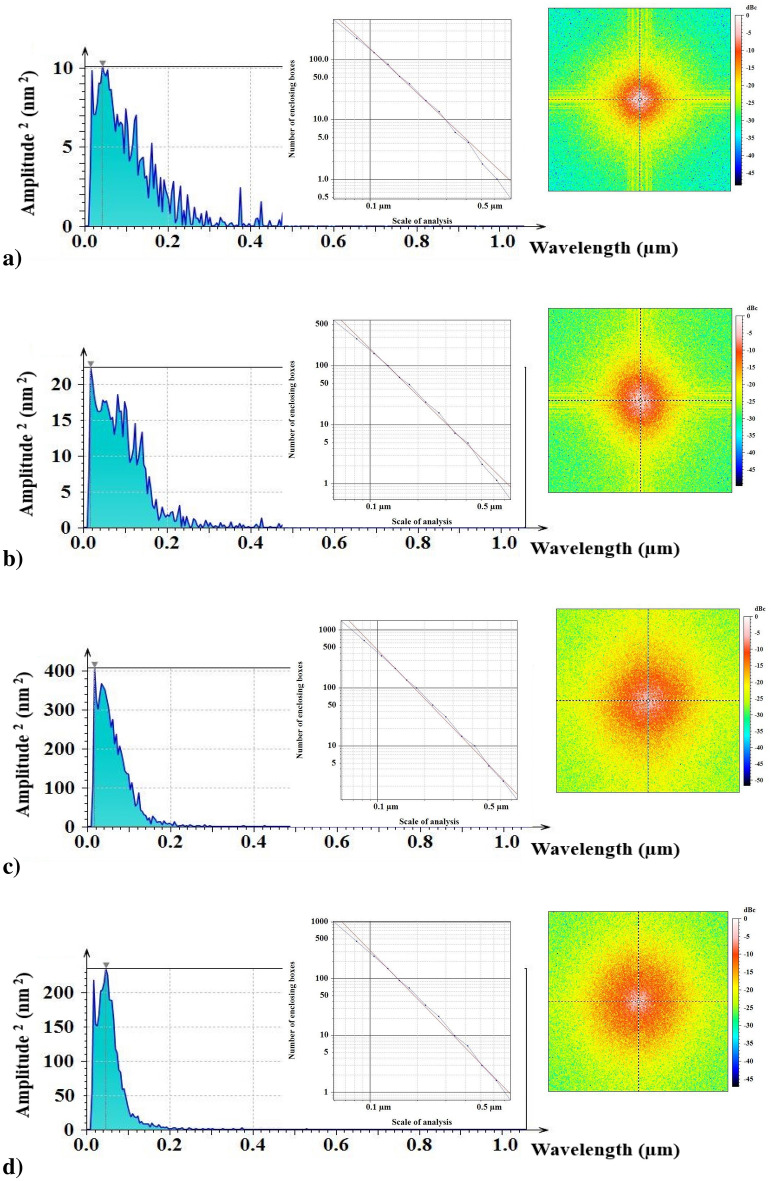
The average PSD of the surface texture, graphical images of the fractal dimension computed by the enclosing boxes method (top left), and frequency spectrum (top right) of the FTO/ZnS thin films annealed at 300, 400, 500, and $550\,^\circ{\text{C}}$.

#### Fractal characterization

**Figure 6:**
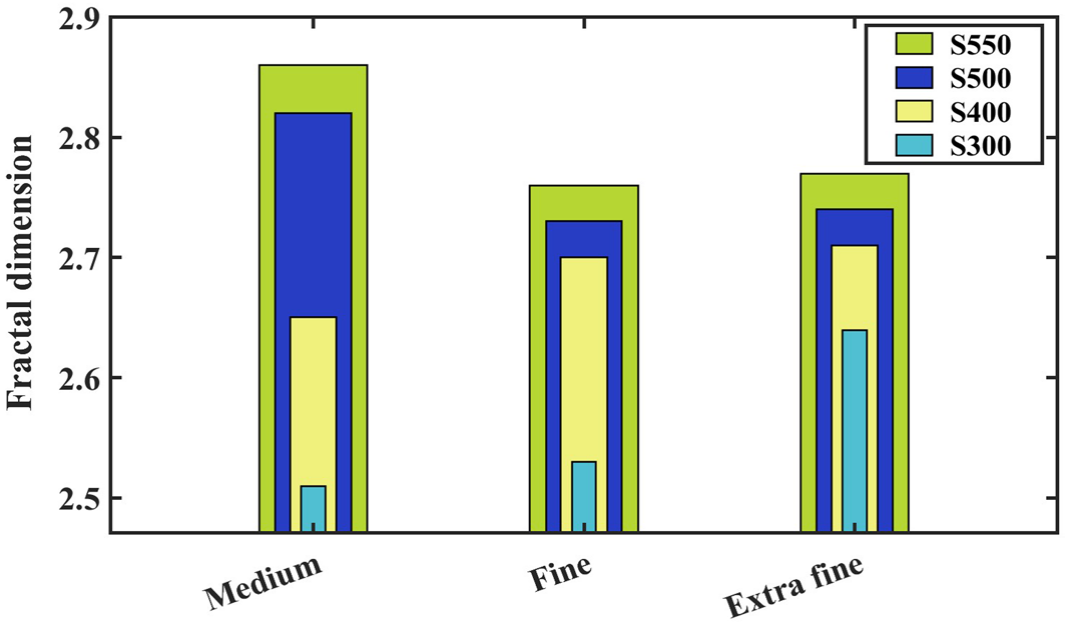
Results of the surface fractal dimension ($D_f$) for three considered options determined by the EBM method for FTO/ZnS thin films annealed at various temperatures.

The power spectral density (PSD) has been a valuable mathematical tool for analysing surface patterns with varying dominant wavelengths. A survey of the PSD involves establishing a correlation between the vertical amplitude and the spatial frequency of surface features. This analytical approach offers valuable insights into the characteristics of the surface microstructure^44^. Figure 5 displays the linearized graphs of the average PSD spectrum. All samples exhibit the same wavelength of $0.5309 \; \upmu{\text{m}}$, maintaining a constant zoom factor of $\times$4. Additionally, sample S500 demonstrated the highest amplitude (0.2082 nm), while sample S300 (0.1457 nm) and sample S550 (0.1822 nm) displayed intermediate values, with the lowest amplitude recorded for sample S400 (0.000 nm). The highest value of the dominant wavelength appears in sample S550 ($0.0467\, \upmu{\text{m}}$), followed by sample S300 ($0.0425 \,\upmu{\text{m}}$). Samples S400 and S500 have the same thickness ($0.0170 \,\upmu{\text{m}}$). The maximum amplitudes are 3.174, 4.737, 20.220, and 15.340 nm for samples S300, S400, S500, and S550, respectively. The fractal dimension, $D_f$, was calculated using the enclosing boxes method (EBM) for extra-fine resolutions, as presented in Fig. 5 (top left). The EBM involves partitioning the area into smaller sections with a width $\varepsilon$ and computing the field $A\varepsilon$ for all the fields covering the entire area. This process is iterative, where the field width is adjusted to plot $ln(A\varepsilon )/ln(\varepsilon )$. A line is fitted to the plot to estimate the fractal dimension by the least squares method. The magnitude of the slope of this fitted line serves as the estimation for the $D_f$. The graph illustrating the calculated volume (for surfaces) is plotted as a function of the scale^45^. The fractal dimension values of the samples were $2.64 \pm 0.08$ (S300), $2.71 \pm 0.08$ (S400), $2.74 \pm 0.08$ (S500), and $2.77 \pm 0.08$ (S550). Generally, a higher $D_f$ in rough fractal surfaces indicates a more complex geometry and irregular shape of the S550 surfaces. The fractal dimension of the sample surfaces was determined using three distinct resolutions, specified by the number of enclosing boxes and the scale of analysis (see Fig. 6). Figure 5 (top right) displays the graphical images of the frequency spectrum. The computed magnitude parameters for the frequency spectrum corresponding to samples S300, S400, S500, and S550 are $-45.02$, $-45.08$, $-51.22$, and $-47.17$ $dB_c$, respectively. The findings revealed a statistically significant difference ($p < 0.05$) for all parameters associated with PSD.

### Advanced fractal analysis

In pursuit of a more quantitative depiction of surface microtexture behavior, we employed the fractal dimension with two novel fractal parameters, namely, fractal succolarity, and lacunarity. In some cases, two images may display the same fractal dimension yet exhibit distinct lacunarity or have identical lacunarity but divergent succolarity. Additionally, a variety of other combinations of results may arise. Fractal socularity (FS) represents the surface penetration and establishes a relationship between the surface texture and the degree of fluid penetration. FS was obtained using the model proposed by N’Diaye et al.^46^:

1 $$\begin{aligned} FS(BS(k), \; dir)= \frac{\sum _{k=1}^n OP(BS(k).\; PR(BS(k), pc))}{\sum _{k=1}^n PR(BS(k), pc))} \end{aligned}$$

where *dir* is the fluid inlet direction (left to right, right to left, top to bottom, and bottom to top), *BS*(*k*) are boxes of equal size with $k \times k$ dimensions, *OP*(*BS*(*k*)) is the occupation percentage, *PR*(*BS*(*k*)) is the occupation pressure, and *pc* is the centroid’s position. Greater succolarity values are associated with diminished percolation, yet when these values approach 0.5, there is an increased likelihood of fluid penetration^47^. When the succolarity is below 0.5, the surface exhibits low percolation. The observed low succolarity values suggest that the roughness might not impact the wettability^48^. The trend of the changes in the contact angle of the samples is consistent with the variations in surface roughness. Therefore, S550 likely presented the highest succolarity value due to its lower porosity. However, the values were close to 0.5, indicating an ideal surface percolation value, as suggested by Melo and Conci^49^ and de Oliveira et al.^47^. Incorporating fractal succolarity and lacunarity can prove valuable for the advanced characterization of surface images^50^. Lacunarity measures the distribution of gaps or voids in a pattern, and the box-counting method is a common approach for characterizing fractal patterns. The calculation of FL follows the model described by Lucena et al.^51^:

2 $$\begin{aligned} FL(r) = \frac{M_2}{{M_1}^2}, \quad M_1(r) = \sum _{s=1}^r s \cdot p(s,r), \quad M_2(r) = \sum _{s=1}^r s^2 \cdot p(s,r) \end{aligned}$$

We derived the lacunarity coefficient ($\beta$) from the lacunarity curve using the following equation to acquire information regarding the surface texture homogeneity^52^:

3 $$\begin{aligned} FL(r)= \alpha . r ^\beta \end{aligned}$$

where *FL*(*r*) is the lacunarity distribution, $\beta$ is the lacunarity coefficient, and *r* is the box size. Lacunarity enables discrimination between sets with identical fractal dimensions but distinct textures^53^. The results summarized in Table 2 show that the lowest value of $\beta$ is observed for sample S300, which suggests that the distribution of gap sizes on the surface is the most uniform^54^. Additionally, the S500 sample exhibited the highest grade of heterogeneity. Lacunarity is indicative of heterogeneity, whereas succolarity signifies anisotropy^55^.

**Table 2:** Advanced fractal parameters of FTO/ZnS thin films.

	S300	S400	S500	S550
*FS*-Fractal Succolarity	$464.6E-3 \pm 49.1E-3$	$471.4E-3 \pm .2E-3$	$476.0E-3 \pm 42.1E-3$	$509.4E-3 \pm 62.4E-3$
$|\beta |$-Lacunarity coefficient	$171.1E-3 \pm 3.6E-3$	$187.6E-3 \pm 4.0E-3$	$184.4E-3 \pm 12.9E-3$	$149.6E-3 \pm 9.9E-3$

Statistically significant difference: *p*-value $$ < 0.05$$

### Wettability behavior

Advanced fractal analysis facilitates understanding the mechanisms of nanostructure formation and provides insights into the wettability of thin film surfaces. This mathematical tool is a decisive parameter governing contact angle dynamics^44^. An increase in wettability can be a consequence of the fractal nature of the surface. The wetting degree of the surface is determined by measuring the contact angle (CA) when a solid and liquid interact. Any solid surface may be wettable(repellent) for a liquid if the contact angle is $> 90^\circ (< 90^\circ$). The effect of annealing temperature on the wettability behavior of the FTO/ZnS bilayer system is studied based on the concept of fractal properties. Regardless of gravity, a drop of liquid on a flat surface assumes the shape of a spherical cap. The interfacial free energy per unit area at the solid-liquid ($\gamma _{sl}$), liquid-vapor ($\gamma _{lv}$), and solid–vapor ($\gamma _{sv}$) interfaces determine the wettability of the surface. The relationship between the contact angle of a liquid droplet with a plane surface and the interfacial free energy is expressed by Young’s basic equation^56^:

4 $$\begin{aligned} cos\theta _Y =\frac{\gamma _{sv} - \gamma _{sl} }{\gamma _{lv}} \end{aligned}$$

Here $\theta _0 =0$ corresponds to complete wetting when the liquid and solid surface energies ($\gamma _{lv}$ and $\gamma _{sv}$) are low and high, respectively. The surface will be hydrophilic (hydrophobic) when the contact angle is > (<) $90^\circ ,$ and $\gamma _{sv}$ > (<) $\gamma _{sl}$^57^. Young’s assumption neglected surface roughness, rendering his model equation incapable of accurately describing wetting behaviors on rough surfaces.^58^. For the first time, Wenzel (in 1936) proposed a theoretical model to describe the contact angle of a liquid drop on a rough solid surface (Young’s modified equation). Wenzel showed that the effective area of a rough solid surface increases by a roughness factor of r (the ratio of the actual area of a rough surface to the projected area)^59^:

5 $$\begin{aligned} r cos\theta _Y =r \frac{\gamma _{sv} - \gamma _{sl} }{\gamma _{lv}} = cos\theta _W \end{aligned}$$

where the contact angle on a smooth surface is denoted by $\theta _Y$ (the Young contact angle), and $\theta _W$ is the apparent contact angle on the rough surface. *r* is called the roughness factor or roughness ratio. As the roughness of the surface increases, the surface becomes more wettable. $r=1$ refers to a smooth surface, while $r\;>1$ corresponds to a rough surface^44^. Originally, surface roughness amplifies the wetting or nonwetting characteristics of a substrate (increasing surface roughness consistently improves either hydrophilicity or hydrophobicity^60^). When water enters a hydrophobic specimen, the increase in roughness leads to an increase in the contact angle increasing the hydrophobicity of the surface^61^. The relationship between the surface fractal dimension $D_f$ and the *r* factor is expressed by a first-order approximation as^62^:

6 $$\begin{aligned} r = \left( \frac{L}{l}\right) ^{D_f-2} \end{aligned}$$

Here, $D_f \;(2< D_f < 3)$ is the fractal dimension, where *L* and *l* denote the upper and lower limit cut-offs/lengths of fractal behavior, respectively, and the associated contact angle corresponding to the classic Wenzel model is expressed as follows^63^:

7 $$\begin{aligned} cos\theta _W= \left( \frac{L}{l}\right) ^{D_f-2} cos\theta _Y \end{aligned}$$

**Figure 7:**
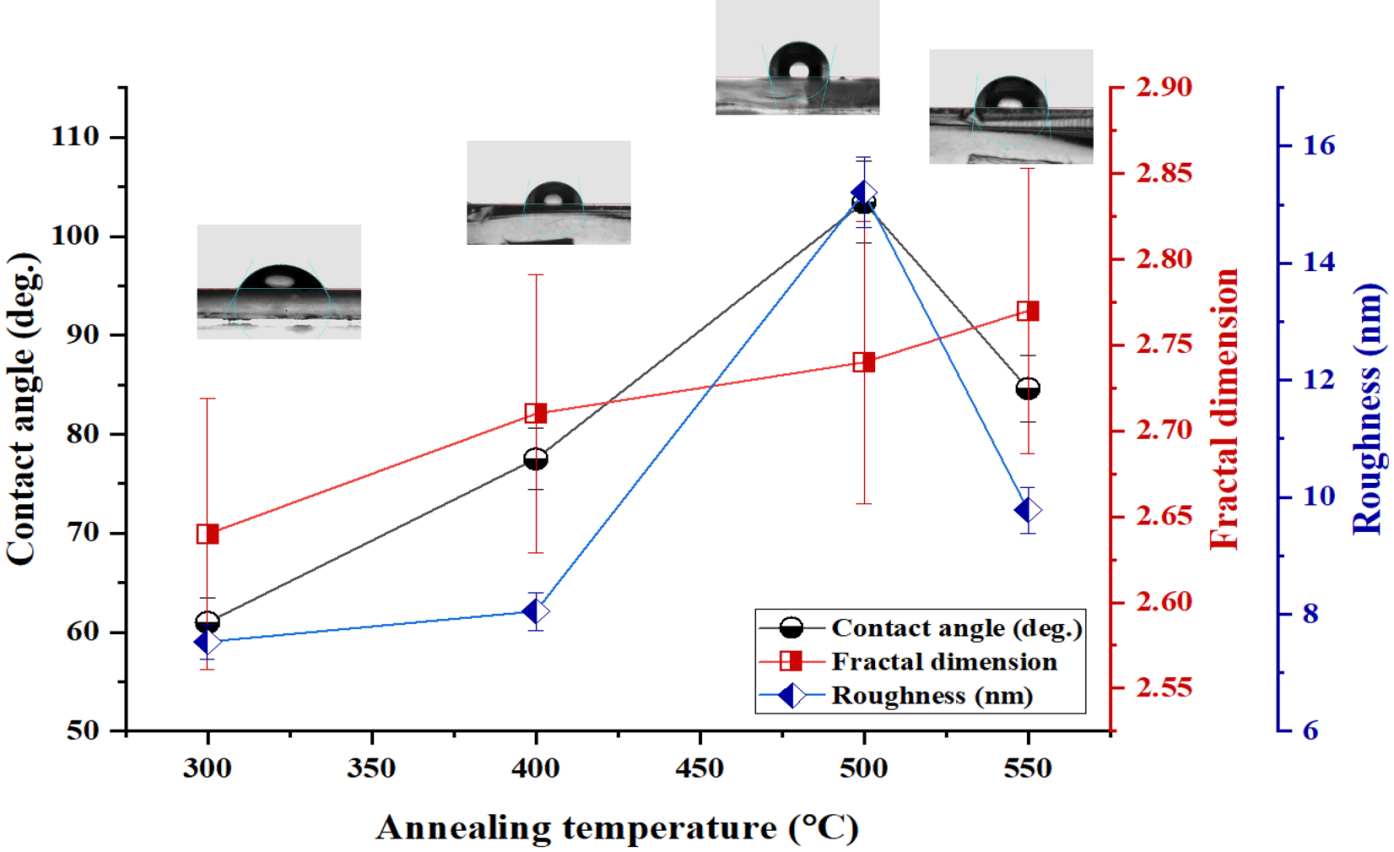
Contact angle, fractal dimension, and roughness of FTO/ZnS thin films versus annealing temperature.

Hence, the surface wettability can be controlled by modifying the fractal dimension, as proposed by Eq. (7). Figure 7 shows the variation in the contact angle, fractal dimension, and roughness with annealing temperature. The observed contact angles for samples S300, S400, S500, and S550 were $61.0 \pm 2.4^\circ$, $77.5 \pm 3.1^\circ$, $103.4 \pm 4.1^\circ$, and $84.6 \pm 3.4^\circ$, respectively. Higher annealing temperatures increase the surface complexity, resulting in increased hydrophobicity. The contact angle measurements suggested that the surfaces of the S500 and S550 samples are hydrophobic, while the surface of the S300 is is highly hydrophilic, as shown in Fig. 7. A surface with a higher fractal dimension will repel water more strongly. As a result, surfaces with higher fractal dimensions yield larger contact angle values. Surfaces with a small fractal dimension do not exhibit significant water-repelling properties, resulting in small contact angle values on such surfaces^64^.

## Conclusions

In conclusion, our study focused on the precise preparation process and characterization of FTO/ZnS bilayer thin films on glass substrates by investigating their 3D-surface morphology, statistical properties, fractal, and wettability behavior. The power spectral density (PSD) is a valuable tool for analysing surface patterns, offering correlations between vertical amplitude and spatial frequency. The calculation of the fractal dimension, employing the enclosing boxes method, exhibited intricate geometries. Topographic characterization through atomic force microscopy provided a detailed understanding of surface morphology from advanced fractal and wettability aspects. We explored the interplay between surface roughness, fractal properties, and wettability, revealing that higher annealing temperatures contribute to increased surface complexity and hydrophobicity. The observed variations indicated a significant impact of annealing temperature on fractal behavior, with higher annealing temperatures leading to increased fractal dimension and fractal succolarity. Following that, as the annealing temperature increases up to $500\,^\circ{\text{C}}$, the increase in roughness points to increasing lacunarity (heterogeneity); further increasing the temperature to $550\,^\circ{\text{C}}$ decreases the roughness and lacunarity. Wettability analysis, through the modified Wenzel equation, employing static water contact angle measurements, revealed a similar trend for hydrophobicity and roughness with annealing temperature, with the highest values of roughness ($15.21 \pm 0.78$ nm) and contact angle ($103.4 \pm 4.1 ^\circ$) obtained at $500\,^\circ{\text{C}}$. F-doped SnO$_2$/ZnS thin films exhibited unique fractal and wettability characteristics, making them highly valuable in applications where surface properties are crucial. Our comprehensive approach, integrating stereometric characterization techniques with advanced fractal analysis, provides a detailed understanding of these bilayer thin films. These findings are valuable for fundamental research in surface science and have practical implications for enhancing hydrophobic coatings, sensor technology, and advanced electronic devices.

## Data Availability

The datasets analysed during the current study are available from the corresponding author upon reasonable request.
